# Molecular Regulation of Catalpol and Acteoside Accumulation in Radial Striation and non-Radial Striation of *Rehmannia glutinosa* Tuberous Root

**DOI:** 10.3390/ijms19123751

**Published:** 2018-11-26

**Authors:** Jingyu Zhi, Yajing Li, Zhongyi Zhang, Chaofei Yang, Xiaotong Geng, Miao Zhang, Xinrong Li, Xin Zuo, Mingjie Li, Yong Huang, Fengqing Wang, Caixia Xie

**Affiliations:** 1 College of Agronomy, Henan Agricultural University, Zhengzhou 450002, China; Emails: jingqing802@126.com (J.Z.); yangcf0204@163.com (C.Y.); 18838916933@163.com (X.L.); zuoxinxinxin@163.com (X.Z.); huangyong16@126.com (Y.H.); 2 School of medicine, Henan University of Chinese Medicine, Zhengzhou 450046, China; LiYajing1365@163.com (Y.L.); gxt0304gg@163.com (X.G.); zm15515889070@163.com (M.Z.); 3 College of Crop Sciences, Fujian Agriculture and Forestry University, Fuzhou 350002, China; zyzhang@fafu.edu.cn (Z.Z.); xinyuzszj@163.com (M.L.)

**Keywords:** tuberous root, catalpol, acteoside, biosynthetic pathway, *Rehmannia glutinosa* L.

## Abstract

*Rehmannia glutinosa* L., a perennial plant of Scrophulariaceae, is one of the most commonly used herbs in traditional Chinese medicine (TCM) that have been widely cultivated in China. However, to date, the biosynthetic pathway of its two quality-control components, catalpol and acteoside, are only partially elucidated and the mechanism for their tissue-specific accumulation remains unknown. To facilitate the basic understanding of the key genes and transcriptional regulators involved in the biosynthesis of catalpol and acteoside, transcriptome sequencing of radial striation (RS) and non-radial striation (nRS) from four *R. glutinosa* cultivars was performed. A total of 715,158,202 (~107.27 Gb) high quality reads obtained using paired-end Illumina sequencing were de novo assembled into 150,405 transcripts. Functional annotation with multiple public databases identified 155 and 223 unigenes involved in catalpol and acteoside biosynthesis, together with 325 UGTs, and important transcription factor (TF) families. Comparative analysis of the transcriptomes identified 362 unigenes, found to be differentially expressed in all RS vs. nRS comparisons, with 143 upregulated unigenes, including those encoding enzymes of the catalpol and acteoside biosynthetic pathway, such as geranyl diphosphate synthase (RgGPPS), geraniol 8-hydroxylase (RgG10H), and phenylalanine ammonia-lyase (RgPAL). Other differentially expressed unigenes predicted to be related to catalpol and acteoside biosynthesis fall into UDP-dependent glycosyltransferases (UGTs), as well as transcription factors. In addition, 16 differentially expressed genes were selectively confirmed by real-time PCR. In conclusion, a large unigene dataset of *R. glutinosa* generated in the current study will serve as a resource for the identification of potential candidate genes for investigation of the tuberous root development and biosynthesis of active components.

## 1. Introduction

*Rehmannia glutinosa* L. is a perennial herb of Scrophulariaceae and is one of the most commonly used herbs in traditional Chinese medicine (TCM) that have been widely cultivated in China. It was recorded in the Chinese medical classic “Shennong’s Herba” and was thought to be a “top-grade” herb in China. In recent decades, many chemical and pharmacological studies have been done on fresh, dried, and steamed tuberous root of *R. glutinosa*. Many natural compounds, including phenylethanoid glycosides (PhGs), iridoid glycosides (IGs), ionone glycosides, polysaccharides, amino acids, and other components, have been found in the herb [1,2,3]. Many studies show that *R. glutinosa* and its active compounds possess wide pharmacological actions on the immune system, the cardiovascular system, the blood system, the endocrine system, and the nervous system [4]. *R. glutinosa* is also thought of as a tonic brain medicine and is commonly used in the prevention and treatment of some central-nervous-system-related diseases, such as depression, cognitive impairment, and anti-anxiety [5].

Extensive research has revealed that the contents of effective components in different organs and tissues of medicinal plants are different. HPLC analysis has revealed that the production of chlorogenic acid, caffeic acid, ferulic acid, luteoloside, and quercitrin is reduced after flowering [6]. Phytochemical analysis demonstrates the localization of tanshinones in the peeled periderm [7] and hydrophilic phenolic acids in the phloem and xylem [8], respectively. The content of major phenolic compounds varies significantly among the olive cultivars and changes during fruit development and maturation, with some compounds showing specificity for certain cultivars [9]. Studies on *R. glutinosa* active compounds accumulation have mainly focused on producing area and harvesting time. For example, the concentration of catalpol in *R. glutinosa* tuberous root is higher in the genuine producing areas [10], and total iridoid glycosides content gradually increases with tuberous root development [11]. There are great differences in acteoside content in *R. glutinosa* tuberous roots and leaves among the different cultivars. Baixuan and Qinhuai are high-acteoside cultivars with a higher content of acteoside in tuberous root [12].

A transverse section of *R. glutinosa* has obvious radial striation from the genuine producing area, which was identified as a sign of genuine medicinal material by the generation of pharmacis. Therefore, the existence of radial striation might be involved in the quality of medicinal materials. In our previous research, we found that the content of catalpol and acteoside of *R. glutinosa* at different development stages varied with different change trends, and there was a significant difference in the component accumulation between radial striation (RS) and non-radial striation (nRS) of tuberous root [13]. However, the molecular mechanism of *R. glutinosa* radial striation and non-radial striation quality formation is still unclear.

Catalpol is an iridoid glycoside, a main active compound of *R. glutinosa*, which possesses many therapeutic effects, including anti-inflammatory effects, the promotion of sex hormone production, a reduction in bleeding, protection against liver damage, and a reduction in elevated blood sugar [4,14]. Acteoside is a phenylpropanoid, which has remarkable bioactivities, including antioxidant, antinephritic, anti-inflammatory, hepatoprotective, immunomodulatory, and neuroprotective effects [15]. Catalpol and acteoside are usually selected as the two quality-control components in the *R. glutinosa* herb and have been included in the Chinese Pharmacopoeia since the 2015 edition. Considering limited genomic information coupled with genome complexity (polyploidy), several reports about the catalpol and acteoside biosynthesis pathway have been seen in some studies [16,17,18], but the molecular regulation mechanism needs to be deeply studied.

Advancements in high-throughput sequencing technology resulted in cost reductions, rendering transcriptome sequencing the most direct and effective way to explore the biosynthesis mechanism of active compounds in medicinal plants. Here, we carried out transcriptome sequencing in *R. glutinosa* RS and nRS tissues to explore the biosynthesis mechanisms of catalpol and acteoside.

## 2. Results

### 2.1. Quality Characteristics of Radial Striation and Non-Radial Striation of R. glutinosa

Radial striation is the main feature of the appearance of the genuine *R. glutinosa*, clearly present in the fresh and dried tuberous root of *R. glutinosa* [13]. It is light white in the fresh tuberous root of *R. glutinosa*, but the non-radial striation part is light yellow with red oil spots (Figure S1A). Meanwhile, the radial striation of dried tuberous root of *R. glutinosa* is grayish white, and another part is grayish yellow (Figure S1B).

**Figure 1:**
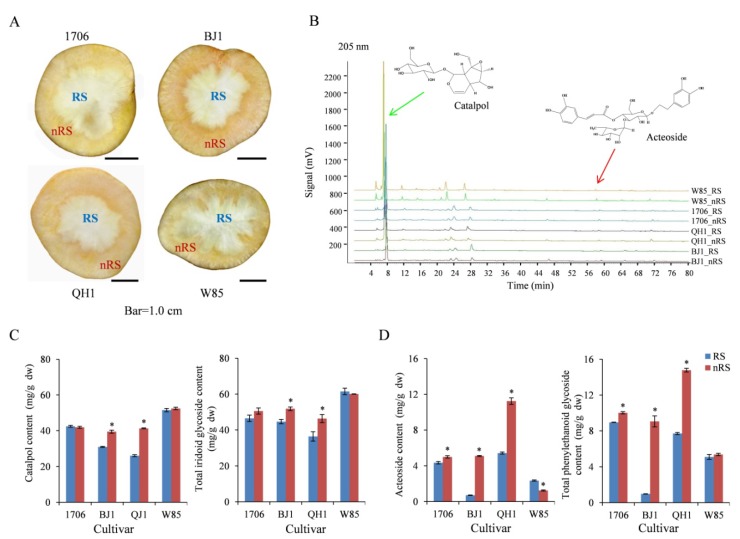
Morphology and active compounds distribution of the *R. glutinosa* tuberous roots. (**A**) Cross sections of tuberous roots from 1706, BJ1, QH1, and W85; (**B**) HPLC chromatographic fierprints in radial striation and non-radial striation of tuberous roots, and monitored at 205 nm, the green arrow and red arrow point to peaks of catalpol and acteoside, respectively; (**C**) the contents of catalpol and total iridoid glycosides in radial striation and non-radial striation; (**D**) the contents of acteoside and total phenylethanoid glycosides in radial striation and non-radial striation. Data shows the average values ± SE of three independent experiments. * indicate significant difference at *p* < 0.05. Abbreviations: RS, radial striation; nRS, non-radial striation. The same as follows.

Different cultivars of *R. glutinosa* have varying medicinal ingredients, and the content presents typical tissue characteristics [12]. In previous studies, we found that there are significantly different medicinal ingredients between radial striation and non-radial striation [13]. By observing the cross-sectional size of the four *R. glutinosa* cultivars, it was found that the proportions of radial striation in 1706 and Wen 85-5) (W85) were larger, and they were smaller in BJ1 and QH1 (Figure 1A). The fingerprint of *R. glutinosa* was then constructed, and the peak of sterol was highest. Furthermore, the content of some chemical components was different in the two parts of tuberous root (Figure 1B). The determination result showed that catalpol and total iridoid glycoside levels were higher in the non-radial striation of BJ1 and QH1 in the middle period of the tuber enlargement (Figure 1C). Simultaneously, acteoside and total phenylethanoid glycoside levels in 1706, BJ1, and QH1 were higher in non-radial striation than those in radial striation, especially in BJ1 and QH1 (Figure 1D). 

In the later period of tuber enlargement, the amount of catalpol in non-radial striation was higher in both BJ1 and W85, and the amount of acteoside in non-radial striation was higher than in radial striation in all four cultivars (Figure S2).

This indicates that the catalpol and other iridoid glycosides are not tissue-specific in some *R. glutinosa* cultivars, while the phenylethanoid glycosides such as acteoside are mainly distributed in non-radial striation. These data can be used to identify candidate genes involved in catalpol and acteoside biosynthesis. 

### 2.2. R. glutinosa Transcriptome Sequencing and Unigene Assembly

For a comprehensive overview of the gene expressing profiles in *R. glutinosa* radial striation and non-radial striation tissues, radial striation and non-radial striation cDNA libraries were constructed, respectively, from the four *R. glutinosa* cultivars, 1706, BJ1, QH1, and W85, and sequenced by the Illumina^TM^ HiSeq 4000 platform in our experiments. The paired-end (PE) sequencing of 24 different libraries resulted in 859,849,100 raw reads, ranging from 33.75 to 37.56 million for each library. Quality filtering after the removal of adaptor sequences, as well as ambiguous and low quality reads, resulted in 715,158,202 high quality reads being obtained (Table 1). After assembling all samples together and filtering the abundance, we obtained 150,405 unigenes. The total length, average length, N50, and GC content of unigenes are 187,170,349 bp, 1,244 bp, 1,974 bp, and 40.87%, respectively (Table S2). 

### 2.3. Functional Annotation of R. glutinosa Unigenes

**Figure 2:**
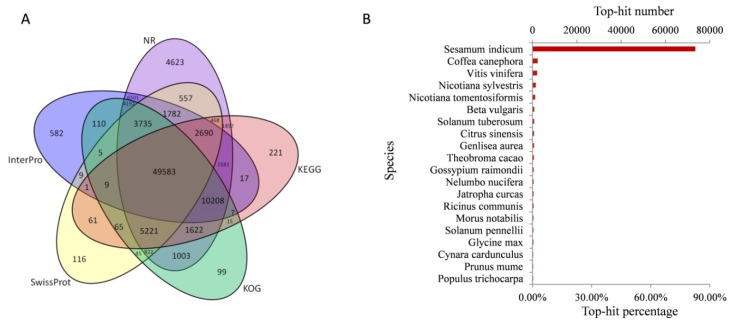
Characteristics of homology search of *R. glutinosa* unigenes. (**A**) Venn diagram of shared and unique unigenes in *R. glutinosa*. Annotation according to the nr, nt, Swiss-Prot, KEGG, COG, GO, and Interpro databases; (**B**) number and percentage of unigenes matching the 25 top species using BLASTx in the nr database.

To provide putative annotations for the unigenes, all of the assembled unigenes were aligned to the protein databases NR, Swiss-Prot, KEGG, KOG, and Interpro using BLASTx and the nucleotide sequence database NT by BLASTn. A total of 103,938 unigenes (69.11% of all 150,405 unigenes) were annotated, including 97,545 in NR, 83,272 in NT, 65,209 in Swiss-Prot, 74,726 in KEGG, 76,781 in KOG, 82,012 in Interpro, and 25,209 in GO (Figure 2A and Table S3). To study the sequence conservation of *R. glutinosa* in other plant species, we analyzed the species distribution of the All-Unigene datasets by aligning sequences against the NR database. The result shows that 75.26% of the distinct sequences have top matches (first hit) with the sequences from *Sesamum indicum*, followed by *Coffea canephora* (2.46%), *Vitis vinifera* (2.2%), *Nicotiana sylvestris* (1.55%), and *N. tomentosiformis* (1.25%) (Figure 2B).

TransDecoder software was used to screen the candidate coding region among the unigenes. The longest ORF (open reading frame) was selected and blast with the SwissProt and Hmmscan database to search for the Pfam protein homology sequence for the prediction of CDS (Coding DNA sequences). In total, more than 102,764 CDS were predicted from All-Unigene sequences (Table S4).

Taken together, All-Unigene represented by far the most comprehensive transcriptome database of *R. glutinosa*, and the dataset was used as the reference transcriptome database in the gene expression analysis.

### 2.4. Expression Analysis for Radial Striation and Non-Radial Striation of Four R. glutinosa Cultivars

**Figure 3:**
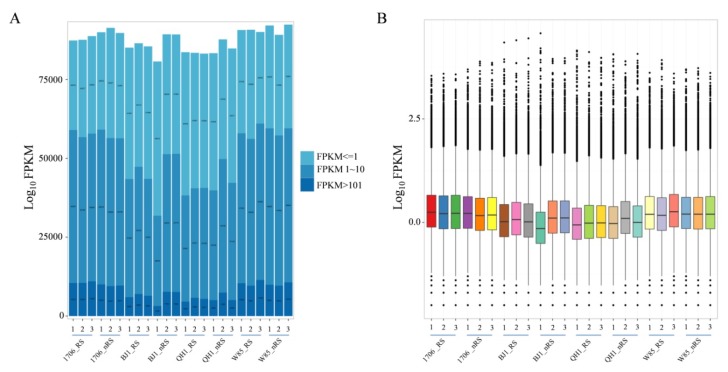
Unigenes expressed number and level on different tissues. (**A**) The number of expressed genes in each sample; (**B**) Expressional levels of unigenes of all the samples. Up and down lines of colored squares indicate upper and lower quartiles, and middle lines indicate medians. Black balls indicate outliers of expression values.

In order to obtain the gene expression profiles of each individual tissue, reads from different tissues were mapped to the obtained non-redundant unigenes. According to the FPKM (fragments per kb per million fragments) values, the mappable reads were used to estimate the transcription levels. Among 24 samples of *R. glutinosa*, more than 60.0% of unigenes both in radial striation and non-radial striation from 1706 and W85 had FPKM values greater than 1, showing a higher number of transcriptionally active unigenes, while radial striation and non-radial striation from BJ1 and QH1 showed a lower number (Figure 3A). However, expression levels of unigenes from BJ1 and QH1 samples showed a higher degree of dispersion than those from the 1706 and W85 samples (Figure 3B).

In order to analyze the global similarities and differences among tissues and cultivars of *R. glutinosa*, PCA and box-whisker plots were conducted. The first four principal components (PCs) accounted for 82.37% of the variation (Figure S3). The 24 samples were divided into different groups according to the PCA loading plot, and six replicates from the same cultivar clustered closely in the same region. However, the radial striation and non-radial striation of the same cultivar were not clustered together (Figure S3). This may be because radial striations and non-radial striations both belong to *R. glutinosa* tuberous root. Most samples from the All-Unigenes database showed expression patterns similar to these tissues.

**Figure 4:**
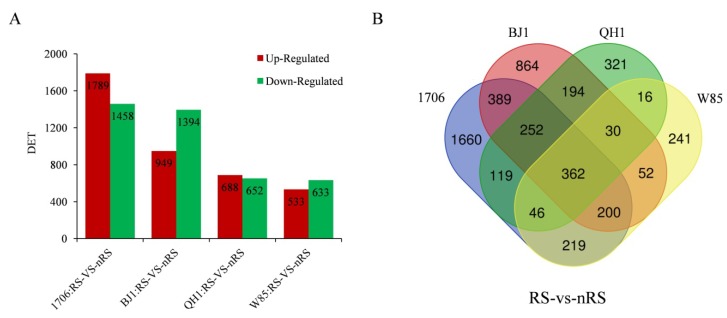
Unigenes significantly differentially expressed between radial striation and non-radial striation in different *R. glutinosa* cultivars. (**A**), The up-regulated and down-regulated transcript numbers in radial striation and non-radial striation. *X* axis represents pairwise and *Y* axis means number of screened DETs. Green bar denotes down-regulated genes and red bar for the up-regulated. (**B**), Venn diagram analysis of the quantity of DETs identified in radial striation and non-radial striation.

To identify *R. glutinosa* genes that were significantly up- or downregulated in non-radial striation compared to corresponding radial striation controls, DeSeq2 was used to generate log_2_ fold change values for each gene. The unigenes that had at least a two-fold change with an Adjusted *p*-value ≤ 0.05 were screened and taken as differentially expressed transcripts (DETs). It was interesting that 1706_RS vs. 1706_nRS comparisons had the most differentially expressed unigenes: 3247 in total, with 1789 upregulated genes and 1458 downregulated genes. Only 1166 DETs were determined between W85_RS vs. W85_nRS, with 533 upregulated genes and 633 downregulated unigenes (Figure 4A). In total, 362 unigenes were found to be differentially expressed in all comparisons. The tissue-specific expression of these unigenes revealed that only 1660, 864, 321, and 241 genes from 1706, BJ1, QH1, and W85, respectively, were found to be differentially expressed in RS vs. nRS comparisons (Figure 4B).

In detail, 143 common upregulated genes were found among the four comparisons, including the phenylalanine ammonialyase gene (*PAL*, *CL2036*.*Contig6*), the geraniol 8-hydroxylase gene (*G10H*, *CL1629*.*Contig4*), and the bHLH transcription factor gene (*CL15084*.*Contig2*) (Figure S4A; Table S5). In contrast, up to 219 common downregulated genes were found in the same comparisons, including the auxin-responsive protein gene (*ARF*, *CL15166*.*Contig1*), the aluminum-activated malate transporter gene (*ALMT*, *Unigene23223*), and the ABC transporter B family member gene (*ABCG*, *CL15718*.*Contig25*) (Figure S4B; Table S6).

**Figure 5:**
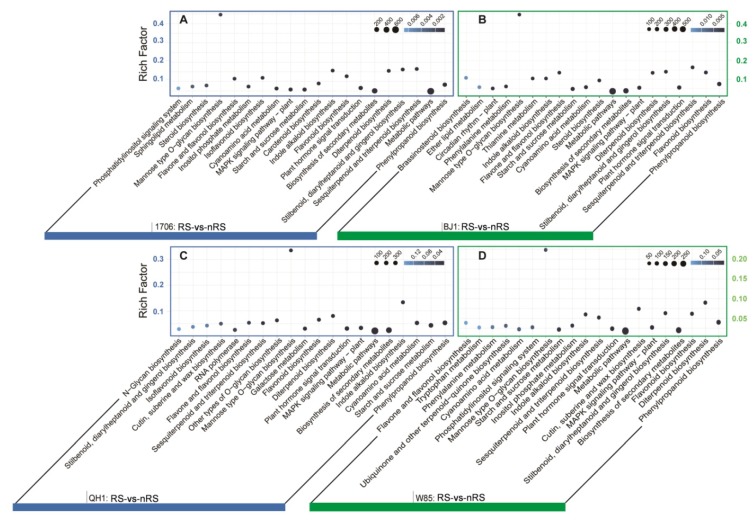
Top 20 enriched KEGG pathways among the annotated DETs across four comparisons. (**A**) Significant enrichment pathways for genes in 1706_RS vs. 1706_nRS comparison. (**B**) Significant enrichment pathways for genes in BJ1_RS vs. BJ1_nRS comparison. (**C**) Significant enrichment pathways for genes in QH1_RS vs. QH1_nRS comparison. (**D**) Significant enrichment pathways for genes in W85_RS vs. W85_nRS comparison. The *X*-axis below represents KEGG pathways, and the *Y*-axis indicates the enrichment factor. Low q-values are shown in blue, and high q-values are depicted in white. Point size indicates DET number (more: big, less: small).

To further identify the functions of the DETs between radial striation and non-radial striation libraries, the KEGG pathway classification was carried out for the functional enrichment of the unigenes. Among these DETs with a KEGG pathway annotation, 2531, 1814, 1044, and 910 DETs were identified in the RS vs. nRS libraries from 1706, BJ1, QH1, and W85, which mapped onto 126, 120, 116, and 112 KEGG pathways (Table S7). To determine whether genes involved in secondary metabolites were enriched, the KEGG pathway database was searched using the DETs to reveal the top 20 significantly enriched pathways. The results showed that 413, 309, 195, and 156 DETs were related to the “biosynthesis of secondary metabolites.” Furthermore, we also noticed that “phenylpropanoid biosynthesis” was the first enriched pathway in all comparisons, the numbers of DETs involved in phenylpropanoid biosynthesis were 148, 134, 95, and 71, respectively. The DETs related to “sesquiterpenoid and triterpenoid biosynthesis” and “diterpenoid biosynthesis” had also been enriched (Figure 5 and Table S7). Beyond that, 22, 12, 10, and 9 DETs were enriched in “terpenoid backbone biosynthesis” (Table S7). The pathway analysis of the DETs between radial striation and non-radial striation libraries could provide valuable information for the following research on the molecular mechanisms of catalpol and acteoside biosynthesis in *R. glutinosa*.

### 2.5. Genes Related to Catalpol Biosynthesis in R. glutinosa

**Figure 6:**
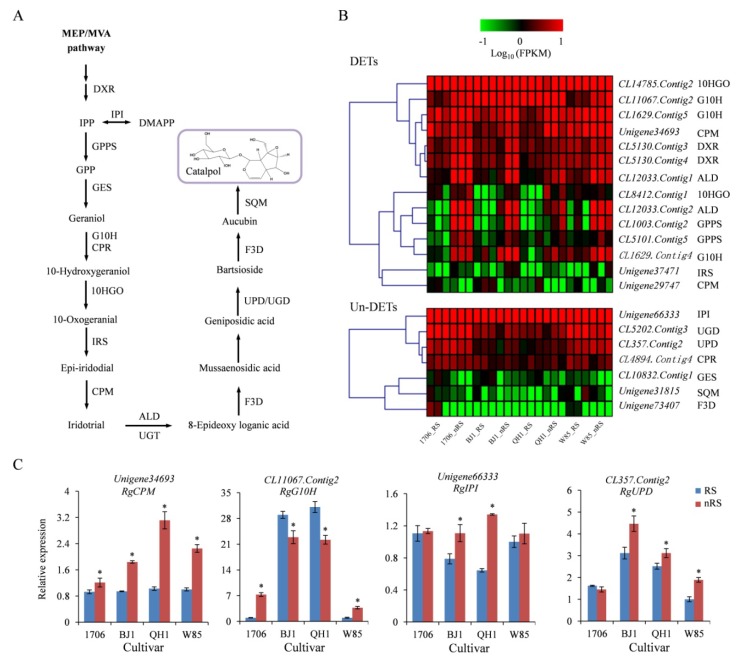
Expression patterns of catalpol biosynthetic unigenes between radial striations and non-radial striations of *R. glutinosa*. (**A**) Biosynthetic pathway of catalpol. (**B**) Heat map representing expression dynamics of unigenes involved in biosynthesis. The expression values (FPKM) for unigenes were log_10_ transformed and scaled across each row, and the heatmap was generated by MultiExperiment Viewer (MeV). (**C**) Relative expression of candidate genes in involved in catalpol biosynthesis. These genes expression levels were all determined by Real-time PCR (qRT-PCR). Vertical bars indicate the standard deviation of three biological replicates. Asterisks (*) indicate a significant difference at the *p* < 0.05 level. Abbreviations: DXR, 1-deoxy-D-xylulose 5-phosphate reductoisomerase; GPPS, geranyl diphosphate synthase; IPI, isopentenyl-diphosphate Delta-isomerase; GES, geraniol synthase; G10H, Geraniol 10-hydroxylase; CPR, NADPH--cytochrome P450 reductase; 10HGO, 10-hydroxygeraniol dehydrogenase; IRS, iridoid synthase; CPM, Cytochrome P-450 monooxygenase; ALD, aldehyde dehydrogenase; UGT, Uridine diphosphate glycosyltransferase; F3D, flavanone 3-dioxygenase; UPD, uroporphyrinogen decarboxylase; UGD, UDP-glucuronic acid decarboxylase; SQM, squalene monooxygenase.

Based on precursor feeding experiments, researchers established the pathway of catalpol biosynthesis and inferred that catalpol and other iridoid glycosides were generated from geraniol, which was produced through a combined biosynthetic route involving non-mevalonate (MEP) and mevalonate (MVA) pathways [19,20]. Shitiz et al. reported that the complete biosynthetic pathway of catalpol has been deciphered for all possible intermediates with their corresponding enzymes in *Picrorhiza kurroa* (Figure 6A) [21]. The putative genes encoding enzymes in the upstream of the iridoid pathway, including geraniol synthase (GES), geraniol 10-hydroxylase (G10H), cytochrome P450 reductase (CPR), and 10-hydroxygeraniol oxidoreductase (10HGO), have been identified in *R. glutinosa* [16]. However, the genes encoding enzymes in the downstream of *R. glutinosa* catalpol biosynthesis are still unknown.

In this study, we identified 155 unigenes encoding 16 enzymes involved in the catalpol biosynthesis of *R. glutinosa*, including 102 unigenes encoding seven enzymes upstream of the iridoid pathway, including 1-deoxy-D-xylulose 5-phosphate reductoisomerase (DXR), geranyl diphosphate synthase (GPPS), isopentenyl-diphosphate Delta-isomerase (IPI), GES, G10H, CPR, and 10HGO. Most interestingly, we also identified 53 unigenes encoding enzymes of the downstream of the catalpol biosynthesis pathway, including iridoid synthase (IRS), cytochrome P-450 monooxygenase (CPM), aldehyde dehydrogenase (ALD), flavanone 3-dioxygenase (F3D), uroporphyrinogen decarboxylase (UPD), UDP-glucuronic acid decarboxylase (UGD), and squalene monooxygenase (SQM), in our *R. glutinosa* transcriptome.

Expression analysis of 155 genes involved in the catalpol biosynthesis pathway was done in radial striation and non-radial striation of *R. glutinosa* tuberous root for the four cultivars. The results showed that 16, 11, 9, and 9 genes were upregulated in 1706, BJ1, QH1, and W85 non-radial striations compared to their radial striations, respectively (Table S8). Four unigenes, *CL1003*.*Contig2* (GPPS), *CL1629*.*Contig4* (G10H), *CL12033*.*Contig1* (ALD), and *CL12033*.*Contig2* (ALD), were upregulated in non-radial striations for every cultivar (Table S8, Figure 6B). *Unigene34693* (CPM) was significantly up-regulated only in non-radial striations of BJ1 and QH1 (Table S8, Figure 6B), which matched the change of catalpol content (Figure 1C). However, the qRT-PCR analysis found *Unigene34693* (CPM) significantly upregulated in non-radial striations of the four cultivars (Figure 6C), which does not agree with digital gene expression profiling (DGE) analysis. Instead, both DGE and qRT-PCR analysis revealed that *CL11067.Contig2* (G10H) was upregulated in non-radial striations in 1706 and W85, but downregulated in BJ1 and QH1 (Table S8, Figure 6C). Interestingly, *Unigene66333* (IPI) was not a DET in transcriptome analysis, but it was shown to be significantly upregulated in non-radial striations of BJ1 and QH1 in qRT-PCR analysis, which is consistent with the changes in catalpol content (Table S8, Figure 6C). In addition, *CL357.Contig2* (UPD), encoding the enzyme catalyze geniposidic acid to bartsioside, was significantly upregulated in non-radial striations of BJ1, QH1, and W85 (Figure 6C). Therefore, the identified candidate catalytic enzyme genes may be involved in the biosynthesis of catalpol and iridoid glycosides and play important roles in their synthesis and accumulation.

### 2.6. Genes Related to Acteoside Biosynthesis in R. glutinosa

**Figure 7:**
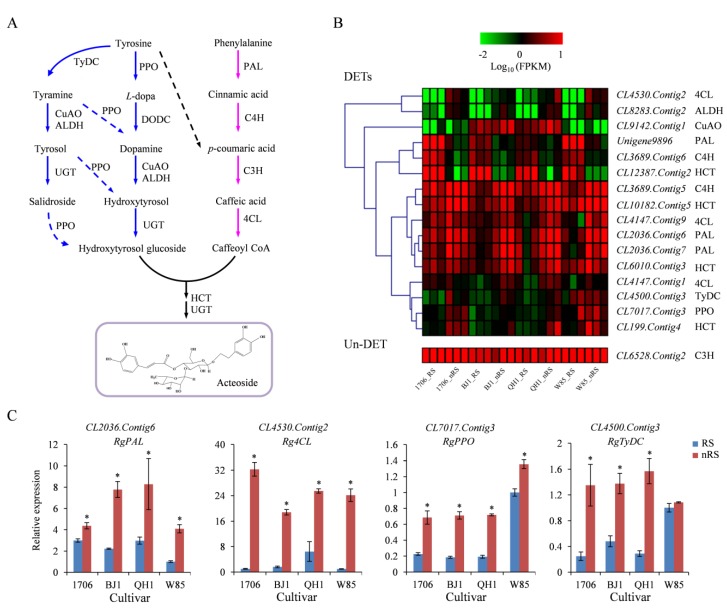
Expression patterns of acteoside biosynthetic unigenes between radial striations and non-radial striations of *R. glutinosa.* (**A**) Biosynthetic pathway of acteoside. The blue arrows indicate the tyrosine-derived pathway, and the purple arrows indicate the phenylalanine-derived pathway. The solid arrows denote known steps and the dashed arrows denote hypothetical steps. (**B**) Expression levels of the identified unigenes were annotated as enzyme coding genes from acteoside biosynthesis pathways. (**C**) Expression levels of unigenes involved in the biosynthesis of acteoside. Vertical bars indicate the standard deviation of three biological replicates. * indicate a significant difference at the *p* < 0.05 level. Abbreviations: PAL: phenylalanine ammonia-lyase; C4H, cinnamate-4-hydroxylase; C3H, coumarate-3-hydroxylase; TyDC, tyrosine decarboxylase; PPO, polyphenol oxidase; CuAO, copper-containing amine oxidase; ALDH, alcohol dehydrogenase; UGT, UDP-glucose glucosyltransferase; 4CL, 4-coumarate-CoA ligase; HCT, Shikimate *O*-hydroxycinnamoyltransferase.

The biosynthetic pathway of acteoside in *Olea europaea* has been characterized [22]. In *R. glutinosa*, two biosynthetic pathways are involved in the biosynthesis of the processor of phenylethanoid glycosides: the phenylalanine-derived pathway and the tyrosine-derived pathway [18] (Figure 7A). In the phenylalanine-derived pathway, phenylalanine is metabolized to caffeoyl-CoA through phenylalanine ammonia-lyase (PAL), cinnamate-4-hydroxylase (C4H), coumarate-3-hydroxylase (C3H), and 4-coumarate-CoA ligase (4CL). In the tyrosine-derived pathway, tyrosine is transformed into hydroxytyrosol glucoside by tyrosine decarboxylase (TyDC)/DOPA decarboxylase (DODC), copper-containing amine oxidase (CuAO), alcohol dehydrogenase (ALDH), polyphenol oxidase (PPO), and UDP-glucose glucosyltransferase (UGT). Caffeoyl-CoA and hydroxytyrosol glucoside were further catalyzed by shikimate *O*-hydroxycinnamoyltransferase (HCT) and UGT to form acteoside.

In total, 223 unigenes encoding nine enzymes involved in acteoside biosynthesis have been identified from the novel *R. glutinosa* transcriptome. RNA-seq analysis revealed that 21, 24, 11, and 15 were DETs in the four RS vs. nRS comparisons, and 13, 12, 10, and 10 were upregulated genes, respectively (Table S9). A PAL gene, *CL2036.Contig6*, was significantly upregulated in non-radial striations for every cultivar. *CL12387.Contig2*, by contrast, was significantly downregulated in non-radial striations of all cultivars (Table S9, Figure 7B). Four unigenes, including *CL3689.Contig5* (C4H), *CL8283*.*Contig2* (ALDH), *CL4530*.*Contig2* (4CL), and *CL2036*.*Contig7* (PAL), were found as DETs in at least three cultivars. It is very interesting that the values of Log_2_ (nRS/RS) of *CL7017*.*Contig3* (PPO) and *CL4500*.*Contig3* (TyDC) in 1706, BJ1, and QH1 were higher than 1, while the Log_2_ Ratio (nRS/RS) value in W85-5 was less than 0.5, which is similar to the change in acteoside between radial striation and non-radial striation among the four cultivars (Table S9, Figure 7B). qRT-PCR analysis showed that the three unigenes, including *CL2036*.*Contig6* (PAL), *CL4530*.*Contig2* (4CL), and *CL7017*.*Contig3* (PPO), were significantly upregulated in non-radial striations of the four cultivars, while *CL4500*.*Contig3* (TyDC) was only significant up-regulated in non-radial striation of the three cultivars, including 1706, BJ1, and QH1 (Figure 7C), which coincides well with the transcriptome analysis. The relative expression status of pathway genes between radial striations versus non-radial striations thus provided a realistic association with the biosynthesis of acteoside and phenylethanoid glycosides.

### 2.7. UDP-Dependent Glycosyltransferases (UGT) Gene 

UDP-dependent glycosyltransferases (UGTs) are pivotal in the process of glycosylation for decorating iridoid glycoside and phenylethanoid glycosides with sugars. Phenylpropanoids are usually glycosylated at the 3-*O* or 7-*O* position, which serves to increase stability and solubility, as well as decrease the potential toxicity of the aglycone precursor [23]. UGTs belong to the largest family (Family 1) of the glycosyltransferase superfamily, and in *Arabidopsis thaliana*, more than 100 UGTs encoding genes have been identified [24,25].

**Figure 8:**
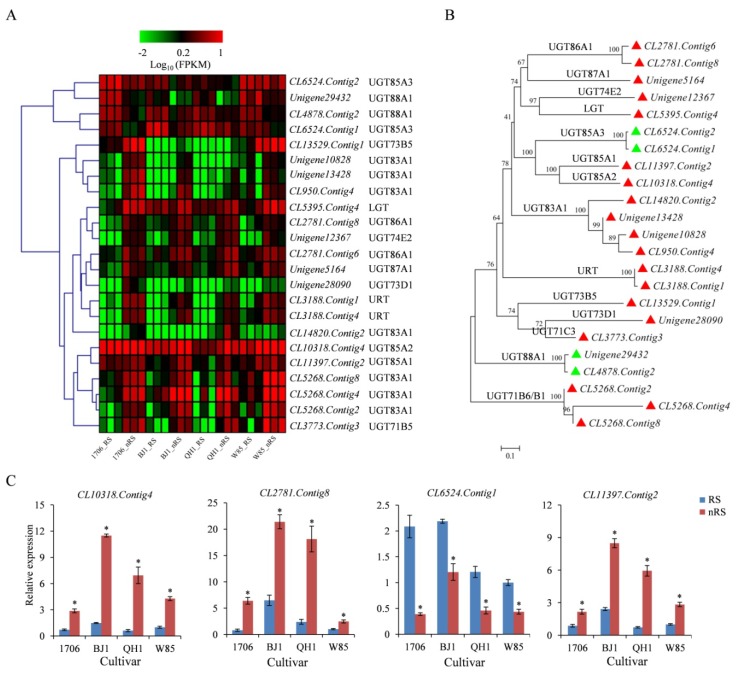
Expression patterns of UGTs between radial striations and non-radial striations of *R. glutinosa.* (**A**) Heat map representing expression dynamics of unigenes belongs to UGT encoding genes. (**B**) Phylogenetic tree of UGT proteins was generated by the neighbor-joining method using software of MEGA 6.0. (**C**) Relative expression levels of four UGT genes in radial striation and non-radial striation from 1706, BJ1, QH1, and W85 by qRT-PCR. Vertical bars indicate the standard deviation of three biological replicates. * indicate a significant difference at the *p* < 0.05 level.

In this research, a total of 325 UGTs were found in the *R. glutinosa* transcriptome. Comparative transcriptome analysis revealed that 32, 25, 12, and 9 unigenes were differentially expressed in non-radial striations compared with radial striations in 1706, BJ1, QH1, and W85. Among them, 27, 23, 11, and 9 were upregulated genes, and only several genes were downregulated. Three members of UGT genes, including *CL3773*.*Contig3*, *CL5268*.*Contig2*, and *Unigene12367*, were upregulated in non-radial striations for every cultivar (Table S10, Figure 8A). Using the deduced amino acid sequences of the 18 *R. glutinosa* UGT genes, multiple amino acid sequence alignment was conducted. The sequence alignment indicated that there is a conserved domain with the sequence of WAP-H-GWNS-E-DQ (Figure S5), which has been reported as a binding domain. Interestingly, homologous comparison analysis found that some UGT genes possessed higher sequence similarities and shared similar expression patterns (Figure 8B). For example, *CL10318*.*Contig4* (UGT85A2) and *CL11397*.*Contig2* (UGT85A1) were clustered into the same group, their expression was significantly upregulated in the non-radial striation of BJ1, and their Log_2_ Ratio(nRS/RS) values were greater than 1 in QH1 (Table S10). The qRT-PCR analysis indicated that *CL10318*.*Contig4* and *CL11397*.*Contig2* have extremely similar expression patterns in detected tissues (Figure 8C). It can be seen from the transcriptome and qRT-PCR analysis that most of the differentially expressed UGT genes showed higher expression levels in non-radial striations of *R. glutinosa* tuberous roots, which provides strong evidence of the molecular mechanisms of active component accumulation.

### 2.8. Transcription Factor Genes 

Transcription factors (TFs) were not only involved in a wide range of plant growth, development, and response to environmental stresses, but also involved in the regulation of the biosynthesis of active ingredients in medicinal plants [26,27,28]. To identify transcription factors involved in the regulation of catalpol and acteoside accumulation in tuberous root, we analyzed transcription factor genes, whose expression was positively or negatively related to catalpol and acteoside content. A total of 4461 unigenes encoding TFs were classified into 59 TF families (Figure S6). The largest gene family was the MYB family, followed by the MYB-related family, the C3H family, the AP2-EREBP family, the bHLH family, the WRKY family, and the ARF family of transcription factors. 

We compared the number of differentially expressed TFs between different groups (Table 2). In all cultivars, the number of differentially expressed TFs was the largest when comparing radial striation to non-radial striation of 1706 for both upregulated and downregulated TFs, while the lowest number of DETs, with 33 upregulated and 50 downregulated TFs, was found for the W85_RS vs. W85_nRS comparison. In total, the DETs of TFs were divided into 27 subfamilies, including WRKY, AP2-EREBP, bHLH, MYB, and so on. 

**Figure 9:**
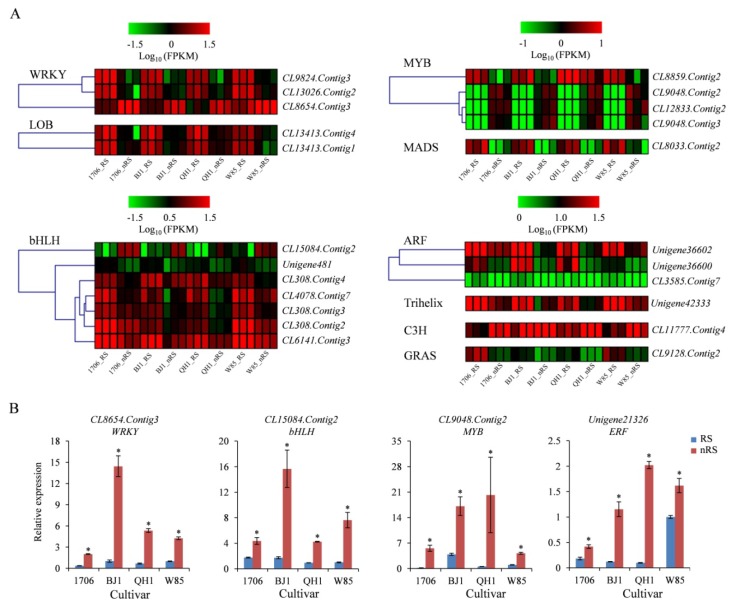
Expression patterns of TFs between non-radial striations than in radial striations of *R. glutinosa.* (**A**) Heat map representing expression dynamics of unigenes belongs to transcription factor genes. (**B**) Relative expression levels of four transcription factor genes in non-radial striations compared to radial striations from 1706, BJ1, QH1, and W85 by qRT-PCR. Vertical bars indicate the standard deviation of three biological replicates. * indicate a significant difference at the *p* < 0.05 level.

The TFs belonging to WRKY, bHLH, MYB, and AP2- EREBP families execute key roles in the regulation of the biosynthesis of secondary metabolites [29,30,31,32]. We identified many differentially expressed WRKY, bHLH, and MYB genes in four comparisons, where only *CL8654.Contig3* (WRKY), *CL15084*.*Contig2* (bHLH), *CL12833*.*Contig2* (MYB), *CL9048*.Contig2 (MYB), *CL9048*.*Contig3* (MYB), and *CL11777*.*Contig4* (C3H) were the upregulated genes, whose expression was significantly higher in non-radial striation than in radial striation (Table S11, Figure 9A). Interestingly, all differentially expressed TFs belong to ARF, and the expression of LOB was significantly lower in non-radial striation than in radial striation. *Unigene21326* (AP2- EREBP) was significantly expressed in the RS vs. nRS comparison for *R. glutinosa* 1706, BJ1, and QH1 (Table S11). However, qRT-PCR analysis revealed that the expression of *Unigene21326* was significantly higher in non-radial striation than in radial striation for all cultivars (Figure 9B). 

## 3. Discussion

The tuberous roots of *R. glutinosa* are derived from the swelling of fibrous roots, which are developed from adventitious roots [33]. It is traditionally believed that each tuberous root consists of two parts: radial striation and non-radial striation [13]. The radial striation is mainly composed of a xylem formed by the proliferation of a large number of parenchyma cells, with a small number of secretory cells. The non-radial striation mainly consists of phloem. The outside has wooden bolt cells and the inside arranges loosely to the parenchyma cells, dispersiving most secretory cells contained in the orange red oil drop, which occasionally has stone cells. Studies have shown that the survival of all vascular plants depends on the xylem and phloem, which comprise a hydraulically coupled tissue system that transports metabolites [34]. In the ancient literature of traditional Chinese medicine (TCM), the radial striation characteristic of *R. glutinosa* tuberous roots is a justification standard for genuine medicinal material. Our previous studies revealed that the active components in the radial striation and non-radial striation of *R. glutinosa* tuberous root were significantly different among different cultivars [35]. However, to date, the molecular regulation mechanisms of quality forming in radial striation and non-radial striation of *R. glutinosa* tuberous root have not been reported.

Chemical composition is an important factor affecting the quality of medicinal materials. However, it is not uniformly distributed in the medicinal materials, as well as the expression of related biosynthesis genes, which all have obvious tissue specificity, such as *Radix polygalae* [36], *Salvia miltiorrhiza* [8], *Lonicera japonica* [37], and *Radix paeoniae* [38]. Therefore, with the rapid development of high throughput sequencing technology, transcriptome sequencing has become an effective method to identify the mechanisms of active component accumulation in medicinal plants.

*R. glutinosa* was one of the earliest medicinal plants to be utilized in high throughput sequencing technology to study the molecular regulation mechanisms of growth and development, stress response, and so on. Using a Solexa DNA sequencing platform, 29 conserved and three novel *R. glutinosa* miRNAs were differentially expressed between the first year of crop (FP) and the second year of crop (SP) plants [39]. Transcriptome was used to identify *R. glutinosa* miRNAs and their targets related to replanting disease [40]. Expression profiling identified a total of 6794 differentially expressed unigenes among *R. glutinosa* adventitious roots (ARs), thickening adventitious roots (TARs), and developing tuberous roots (DTRs), which provided insights into the molecular mechanisms underlying tuberous root development [41]. However, the number of unigene of *R. gutinosa* varied in different experiments. The quality of reference transcriptome may significantly impact the accuracy of expression profile analysis. The power to detect a transcript depends on its length and abundance in the sequencing library, which is affected by library preparation [42]. Li et al. initially obtained 94,544 and 99,708 unigenes from leaf and root transcript libraries, respectively [40]. They then collected 10 root samples from SP (five stages) and FP (five stages) and mixed them together to construct a root transcriptome library, collected 10 leaf samples to construct a leaf transcriptome library, and obtained 71,922 and 54,722 unigenes from the mixed root and mixed leaf libraries via the Illumina HiSeq^™^ 2500 system, respectively [43]. Sun et al. generated a total of 81,003 unique sequences from the cDNA library constructed from the ARs, TARs, and DTRs of *R. glutinosa* by using the Illumina sequencing platform [41]. In this study, we obtained 38,678~54,378 unigenes from the 24 cDNA libraries of radial striations and non-radial striations of *R. glutinosa* tuberous roots and eventually assembled a final set of 150,405 entries with an average length of 1244 bp higher than mixed roots unigenes (383 bp and 678 bp) [17,41] and mixed leaf and root unigenes (868 bp) [43]. This novel unigene database provides a high-quality reference transcriptome on gene expression detection and molecular mechanisms for *R. glutinosa*. 

Gene expression analysis has been extensively utilized for the identification of enzyme genes involved in secondary metabolites biosynthesis by measuring transcriptional levels in different tissues and developmental stages. Phytochemical analysis of the peeled *Salvia miltiorrhiza* root tissues demonstrated the localization of tanshinones in the periderm, and transcriptome analysis revealed that the two key enzyme coding genes *SmCPS1* and *SmKSL1* were specifically expressed in the periderm [7]. Lithospermic acid B accumulates in the phloem and xylem of *S. miltiorrhiza* roots, in agreement with the expression patterns of the identified key genes, including *SmRAS*, *SmC4H1*, and *SmCYP98A78*, related to rosmarinic acid biosynthesis [8]. We identified 155 transcripts (encoding 14 enzymes) involved in the iridoid glycoside biosynthesis pathway from this *R. glutinosa* unigene database. The expression patterns of *Unigene66333* (IPI) and *Unigene34693* (CPM) could match the change in catalpol content and were considered to be the better candidate genes for catalpol biosynthesis. Among the 223 unigenes encoding nine enzymes involved in acteoside biosynthesis, the expression levels of *CL7017*.*Contig3* (PPO) and *CL4500*.*Contig3* (TyDC) in different tuberous root tissues were similar to content changes in acteoside and total phenylethanoid glycosides. Furthermore, *CL4500*.*Contig3* (TyDC) was considered to be the best candidate for acteoside synthesis. Glycosyltransferases (GTs) comprise a ubiquitous family of enzymes that catalyze the transfer of a sugar moiety from an activated donor molecule onto saccharide or non-saccharide acceptors, resulting in the formation of various glycosides of non-carbohydrate moieties [44], which play important roles in acteoside and catalpol biosynthesis. In total, 325 UGTs were identified in this report. Transcriptome analysis showed that three members of UGTs, including *CL3773.Contig3*, *CL5268*.*Contig2,* and *Unigene12367*, were upregulated in non-radial striations for every cultivar. Furthermore, *CL10318.Contig4* and *CL11397*.*Contig2* indicated higher expression levels in non-radial striation of BJ1 and QH, which is consistent with the change in catalpol accumulation, possibly involved in catalpol synthesis. 

Over the past decade, TFs have been key regulators in controlling gene expression by binding to the promoter of single or multiple genes. Many experiments have confirmed that TFs are involved in the biosynthesis of various secondary metabolites in plants [30,32,45,46]. Two bHLH family genes, *TSAR1* (Triterpene Saponin biosynthesis Activating Regulator1) and *TSAR2*, regulate triterpene saponin biosynthesis in *Medicago truncatula* [47]. Overexpression and RNAi transient assays revealed that the two MYB transcription factors (*bMYB10b* and *PbMYB9*) regulate flavonoid biosynthesis in *Pyrus bretschneideri* fruit [30]. Both the qRT-PCR and protein interaction network results showed that four bHLHs (*FabHLH25*, *FabHLH29*, *FabHLH80*, and *FabHLH98*) are responsive to the fruit anthocyanin biosynthesis in strawberries [32]. A total of 4461 transcripts encoding TFs were identified in our *R. glutinosa* novel transcriptome database. Transcriptome analysis revealed that many differentially expressed TF genes showed a higher expression in non-radial striations of *R. glutinosa* tuberous roots. Among them, a member of the ERF family, *Unigene21326*, possibly the key regulator in acteoside biosynthesis, was found to have an expression that was significantly upregulated based on an RS vs. nRS comparison for *R. glutinosa* 1706, BJ1, and QH1.

## 4. Materials and Methods

### 4.1. Plant Materials and Growth Conditions

All samples were collected from the planting base of *R. glutinosa* in Wen County, Henan Province, China (35^°^3′31′′ N, 113^°^7′17′′ E), with a total of four cultivars: W85-5 (W85), BJ1, QH1, and 1706. All materials were sowed in mid-April 2017 by planting a double line at the width ridge. The height, width, and spacing of ridges were 15, 40, and 30 cm, respectively. In addition, all plants of the four cultivars were managed uniformly. Then, samples were taken on September 30, and the radial striation was separated from the non-radial striation by scalpels. In addition, samples used for transcriptome sequencing were stored at −80°C, and the materials for analysis of the quality characteristics were placed in a desiccator after drying and powdering.

### 4.2. Analysis of Quality Characteristics of Radial Striation and Non-Radial Striation of R. glutinosa 

The HPLC fingerprint method established by the research group was used to analyze the samples [13]. The catalpol and acteoside content in the radial striation and non-radial striation of *R. glutinosa* tuberous root was determined by referring to the method of Chinese Pharmacopoeia (2015 edition). Total phenylethanoid glycoside and iridoid glycoside content was determined with a UV spectrophotometer, with reference to the method established by the previous studies [11,48]. 

### 4.3. RNA Isolation and Illumina Sequencing

Total RNA was extracted from the three replicates for the radial striation and non-radial striation of *R. glutinosa* tuberous roots with TRIzol reagent, with reference to the manufacturer’s instructions. RNA quality and quantity were determined with a Nanodrop^TM^ 2000 spectrophotometer (Thermo Fisher Scientific, Waltham, MA, USA) and a Bioanalyzer 2100 (Agilent, California, USA). A total of 24 RNA samples were of sufficient quality with RNA integrity number (RIN) values ranging from 8.0 to 9.5. Then, they were converted to cDNA by reverse transcription, and the cDNA library was sequenced using an Illumina Hiseq^TM^ 4000 system from BGI-Tech (Shenzhen, China; Project ID: F17FTSCCKF3479). 

### 4.4. Sequence Assembly and Annotation

The data obtained by sequencing is known as raw reads or raw data. Quality reads are performed on the raw reads to determine whether the sequencing data is suitable for subsequent analysis. Fragments were assembled using Trinity software after QC was qualified [49]. The assembled Unigene was compared with the seven databases, nr (http://www.ncbi.nlm.nih.gov/, NCBI non-redundant protein sequences) (access on 9 November 2017), nt (NCBI nucleotide sequences), Pfam (Protein family, http://pfam.sanger.ac.uk/) (access on 9 November 2017), KOG (euKaryotic Ortholog Groups, http://www.ncbi.nlm.nih.gov/cog/) (access on 9 November 2017), Swiss-Prot (a manually annotated and reviewed protein sequence database, http://www.expasy.ch/sprot/) (access on 9 November 2017), KEGG (Kyoto Encyclopedia of Genes and Genomes, http://www.genome.jp/kegg/) (access on 9 November 2017), and GO (Gene Ontology, http://www.geneontology.org/) (access on 9 November 2017), by the BLAST program for sequence similarity analysis and functional annotation of unigenes, and the DETs were screened out according to the expression level of genes.

### 4.5. Differential Gene Expression Analysis

Gene expression levels were calculated based on the numbers of reads mapped to the reference sequence using the FPKM method [50]. Differentially expressed genes were identified based on the method described by Audic and Claverie [51]. The differentially expressed genes were defined by default as genes with an FDR ≤ 0.05 and multiple differences of more than two-fold. 

### 4.6. Quantitative RT-PCR Analysis

The expression levels of all genes of interest in the study were detected by quantitative real-time PCR (qRT-PCR). Total RNA was extracted from plant samples with the Plant RNA Extraction Kit (Takara, Dalian, China), according to the kit’s instructions. cDNA was synthesized from about 1 μg of total RNA using Super Script^TM^ reverse transcriptase (Takara, Dalian, China). The amplification reaction of qPCR was performed in a Bio-Rad iQ5 Real-Time PCR System (Bio-Rad, California, USA), using SYBR^®®^ Premix Ex Taq™ II (Tli RNaseH Plus) (Takara, Dalian, China), according to the manufacturer’s instructions. Primers for qRT-PCR analysis are listed in Table S1, and the PCR amplification conditions were as follows: 94 °C for 5 min; 45 cycles of 94 °C for 10 s, 55 °C for 15 s, and 72 °C for 20 s. For each candidate gene, the PCR reactions were carried out in triplicate. The *RgTIP41* gene (GenBank accession number KT306007) was used as internal control to calculate relative expression levels based on the 2^−ΔΔ*C*_t_^ method [52]. 

### 4.7. Data Submission

The transcriptome data of the twenty-four *R. glutinosa* samples were submitted to the GenBank-SRA repository under the project identification number PRJNA504376.

## 5. Conclusions

In this study, we detected the content distribution of catalpol, acteoside, total iridoid glycosides, and total phenylethanoid glycosides in radial striation and non-radial striation of four cultivars: 1706, BJ1, QH1, and W85. Catalpol and total iridoid glycosides amounts were higher in the non-radial striation of BJ1 and QH1, and acteoside and total phenylethanoid glycoside amounts in 1706, BJ1, and QH1 were higher in non-radial striation than that in radial striation. The radial striation and non-radial striation cDNA libraries were constructed, respectively, from the four *R. glutinosa* cultivars and sequenced by the Illumina^TM^ HiSeq 4000 platform. A total of 715,158,202 high quality reads were obtained, and 150,405 unigenes were finally assembled. Among them, 3247, 2343, 1340, and 1166 unigenes were differentially expressed in RS vs. nRS comparisons of 1706, BJ1, QH1, and W85, respectively. The expression profiles of some catalytic enzyme genes and transcript factor genes were positively correlated with content changes of catalpol, acteoside, total iridoid glycosides, and total phenylethanoid glycosides. It will help not only to elucidate the molecular mechanisms of catalpol and acteoside biosynthesis, but also to illuminate the influence of radial striation on the quality characteristics of *R. glutinosa* tuberous roots.

## Figures and Tables

**Table 1:** Summary of the sequencing data from different *R. glutinosa* samples.

Sample	Total Raw Reads	Total Clean Reads	Total Clean Bases (Gb)	Clean Reads Q20 (%)	Clean Reads Q30 (%)	Clean Reads Ratio (%)
1706_RS1	35,930,376	29,939,804	4.49	97.89	93.55	83.33
1706_RS2	35,930,108	30,066,360	4.51	97.91	93.56	83.68
1706_RS3	35,930,118	30,416,510	4.56	97.91	93.6	84.65
1706_nRS1	35,930,582	30,106,740	4.52	97.98	93.78	83.79
1706_nRS2	35,931,096	29,693,676	4.45	98.1	93.9	82.64
1706_nRS3	34,205,142	28,499,086	4.27	97.91	93.59	83.32
BJ1_RS1	35,931,216	29,457,812	4.42	97.91	93.35	81.98
BJ1_RS2	35,931,386	29,683,162	4.45	97.81	93.18	82.61
BJ1_RS3	35,931,264	29,768,646	4.47	97.86	93.27	82.85
BJ1_nRS1	35,931,542	29,720,528	4.46	98.16	94.02	82.71
BJ1_nRS2	35,930,546	29,537,004	4.43	97.83	93.39	82.21
BJ1_nRS3	35,930,574	29,910,368	4.49	97.82	93.36	83.24
QH1_RS1	35,930,624	29,870,448	4.48	97.86	93.42	83.13
QH1_RS2	35,929,752	29,930,562	4.49	97.59	92.8	83.3
QH1_RS3	35,930,062	29,617,920	4.44	97.74	93.13	82.43
QH1_nRS1	35,930,688	29,542,912	4.43	97.8	93.29	82.22
QH1_nRS2	37,564,468	30,470,168	4.57	98.07	93.81	81.11
QH1_nRS3	37,564,500	30,466,862	4.57	98.05	93.69	81.11
W85_RS1	35,930,236	30,246,190	4.54	97.9	93.56	84.18
W85_RS2	35,930,102	30,523,230	4.58	97.95	93.69	84.95
W85_RS3	35,930,692	30,136,144	4.52	97.9	93.59	83.87
W85_nRS1	33,750,468	28,498,496	4.27	97.91	93.62	84.44
W85_nRS2	35,716,050	29,601,754	4.44	97.88	93.52	82.88
W85_nRS3	34,297,508	29,453,820	4.42	97.98	93.78	85.88

**Table 2:** Regulated transcription factors (TFs) between radial striation and non-radial striation in four *R. glutinosa* cultivars.

Transcription Factor	Total of Unigene	1706		BJ1		QH1		W85		All	
		Up	Down	Up	Down	Up	Down	Up	Down	Up	Down
WRKY	184	17	11	11	10	6	8	4	11	1	2
TCP	39	2	2	1	2	1	0	1	1	0	0
MADS	119	3	2	0	2	1	3	0	1	0	1
C2C2-GATA	62	2	1	1	0	0	0	1	0	0	0
AP2-EREBP	286	35	9	17	8	6	2	2	1	0	0
C2H2	93	4	5	1	2	0	2	1	1	0	0
bHLH	235	17	28	9	25	3	16	5	8	1	6
MYB	513	19	21	15	24	9	6	8	3	3	1
Trihelix	104	0	2	0	2	0	1	0	1	0	1
HSF	53	1	1	1	1	0	0	1	0	0	0
C2C2-CO-like	26	1	2	0	1	0	0	0	0	0	0
NAC	141	2	16	1	3	1	0	0	8	0	0
C3H	299	10	2	2	1	3	0	2	0	1	0
C2C2-Dof	94	2	2	0	7	1	0	0	1	0	0
ARF	155	0	15	0	9	0	8	0	5	0	3
FAR1	139	1	0	0	0	3	0	0	2	0	0
SBP	91	9	0	1	1	1	0	3	0	0	0
ABI3VP1	120	1	1	0	0	0	0	1	1	0	0
LOB	49	0	6	0	7	0	6	0	3	0	**2**
FHA	61	0	1	0	0	0	1	0	0	0	0
BES1	47	1	2	1	5	1	3	0	0	0	0
zf-HD	45	0	2	0	1	0	1	0	0	0	0
EIL	22	0	2	0	1	0	0	0	0	0	0
OFP	30	8	1	4	0	0	0	3	0	0	0
GRAS	154	8	2	0	3	0	1	0	2	0	1
Bzip	40	0	2	0	4	0	0	0	1	0	0
TCP	39	2	1	1	0	1	0	1	0	0	0
Total of DET		145	139	66	119	37	58	33	50	6	17

## Supplementary Materials

Supplementary materials can be found at http://www.mdpi.com/1422-0067/19/12/3751/s1.

## Abbreviations

TCM	Traditional Chinese medicine
RS	Radial striation
nRS	Non-radial striation
TF	Transcription factor
UGTs	UDP-dependent glycosyltransferases
PhGs	Phenylethanoid glycosides
IGs	Iridoid glycosides
PE	Paired-end
KEGG	Kyoto Encyclopedia of Genes and Genomes
NT	NCBI nucleotide sequences
GO	Gene Ontology
KOG	EuKaryotic Ortholog Groups
ORF	Open reading frame
CDS	Coding DNA sequences
FPKM	Reads Per Kilobase of exon model per Million mapped reads
DETs	Differentially expressed transcripts
FDR	False discovery rate
