# Supplementary material for: Molecular Regulation of Catalpol and Acteoside Accumulation in Radial Striation and non-Radial Striation of Rehmannia glutinosa Tuberous Root
Source: Int J Mol Sci. 2018 Nov 26;19(12):3751. doi: 10.3390/ijms19123751 (PMC6321003; doi:10.3390/ijms19123751)
Supplement: Supplementary file 1 [file ijms-19-03751-s001.zip › Supplementary Information-Figures.docx]

**Supplementary information**


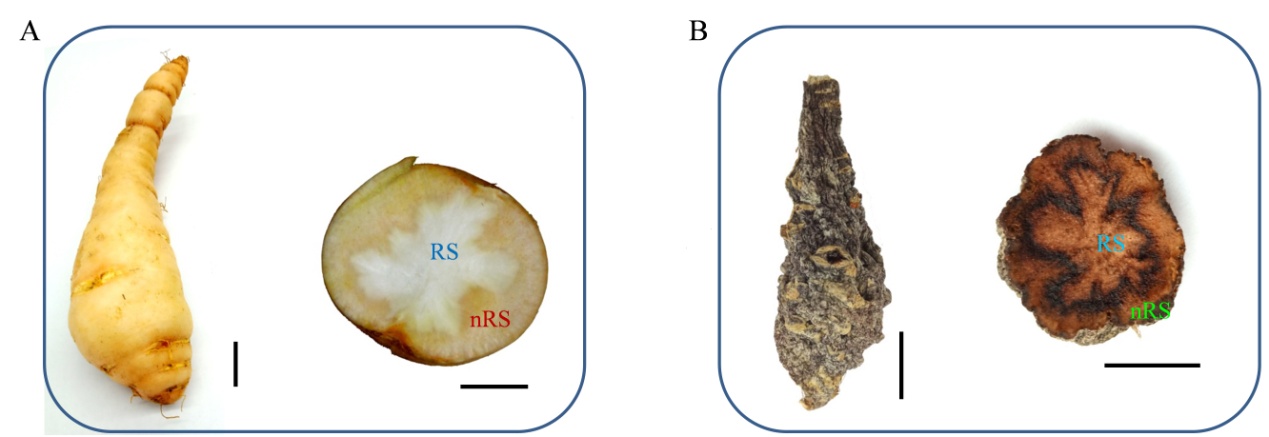


**Figure S1** Feature of the fresh and dried tuberous root of *R glutinosa*. A, Fresh tuberous root of and its cross section; B, Dried tuberous root and its cross section. RS, radial striation; nRS, non-radial striation. Bars are 1.0 cm. Abbreviations: RS, radial striation; nRS, non-radial striation. The same as follows.

**
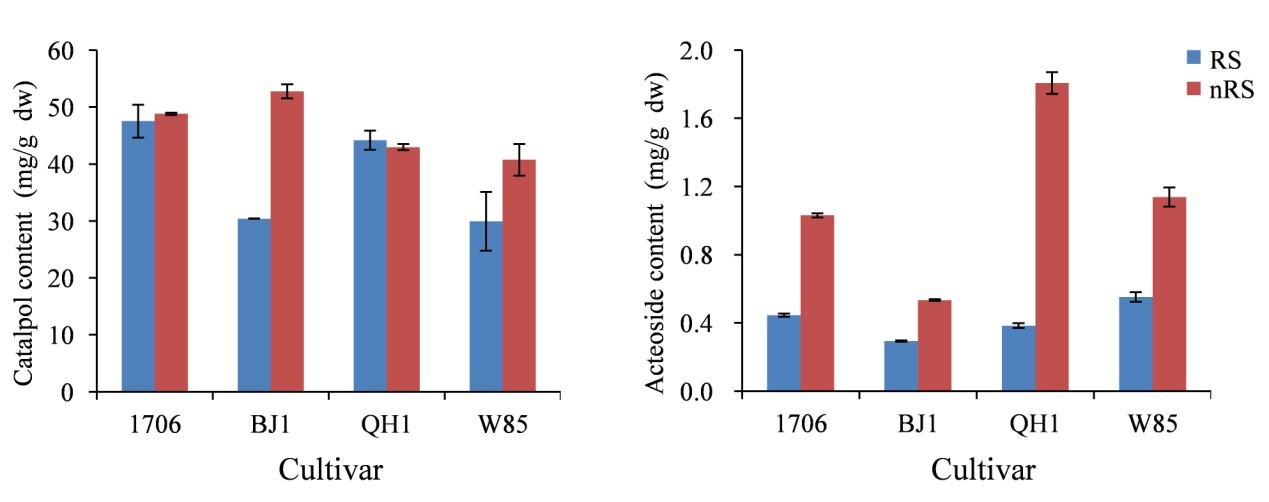
**

Figure S2 Contents of catalpol and total acteoside in radial striation and non-radial striation of *R. glutinosa*

**
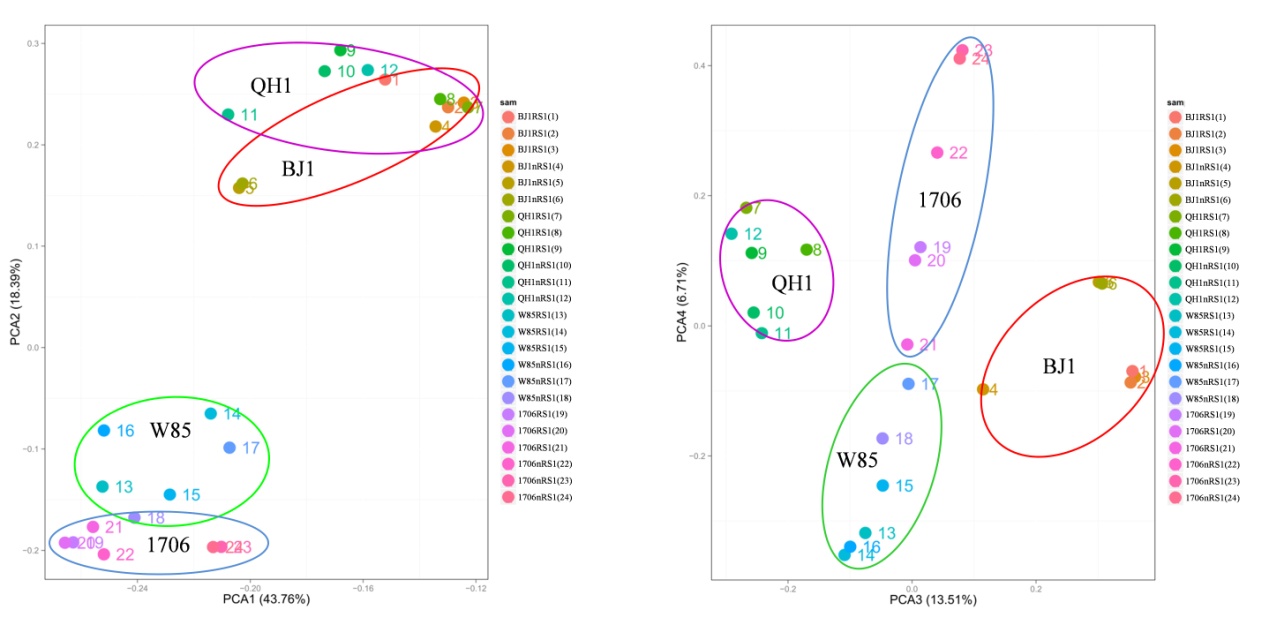
**

**Figure S3** Principal component analysis (PCA) score plots of radial striation and non-radial striation from four *R. glutinosa* cultivars.


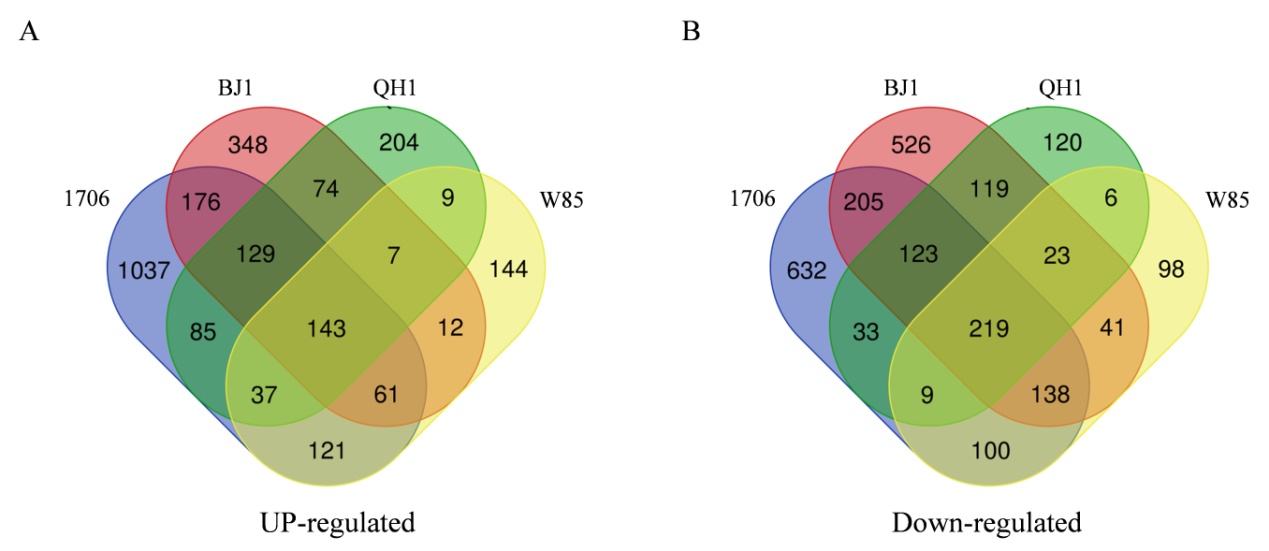


**Figure S4** Venn diagram analysis of the quantity of the up-regulated (A) and down-regulated (B) DETs identified in radial striations and non-radial striations.

**
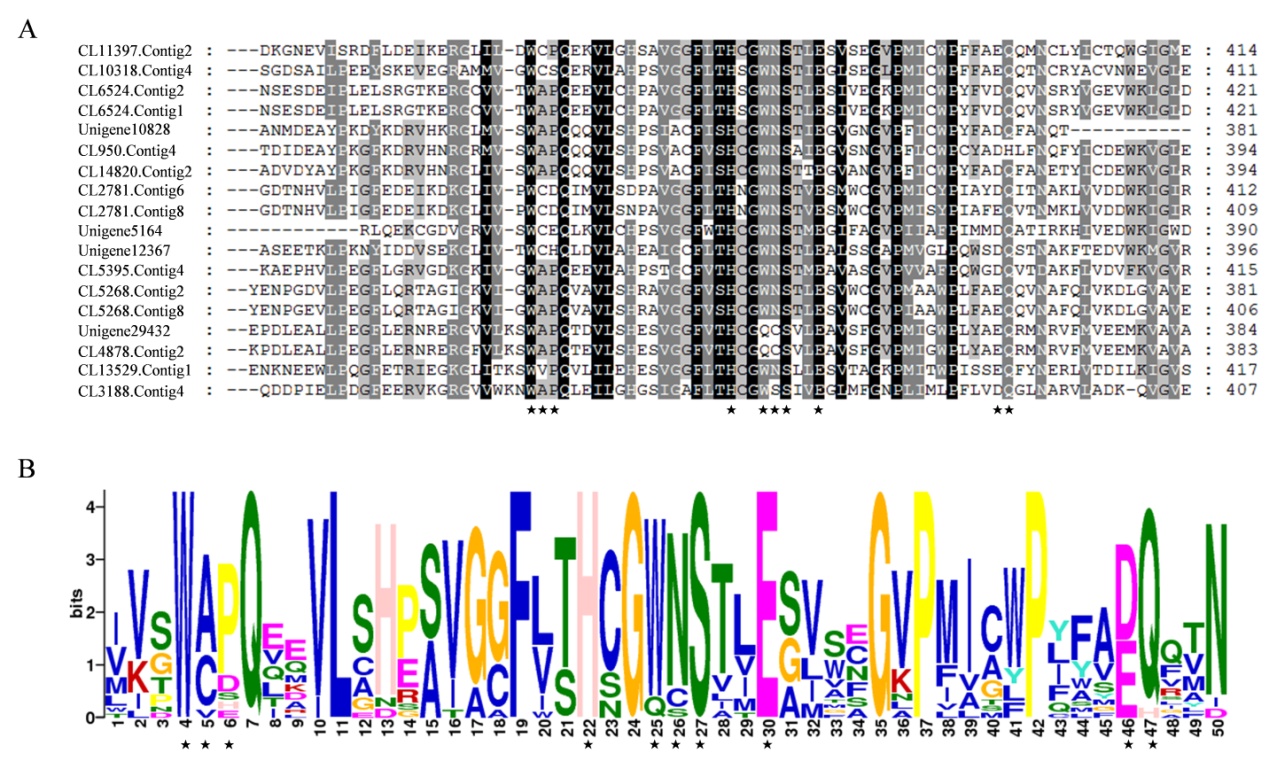
**

**Figure S5** Conserved sequences of *R. glutinosa* UGTs binding domain. A, Conserved amino acid residues are present among the 18 *R. glutinosa* UGTs. The highly conserved amino acid residues in each repeat are highlighted in dark gray. B, Sequence logos of the 18 UGTs with UGT binding domain. **
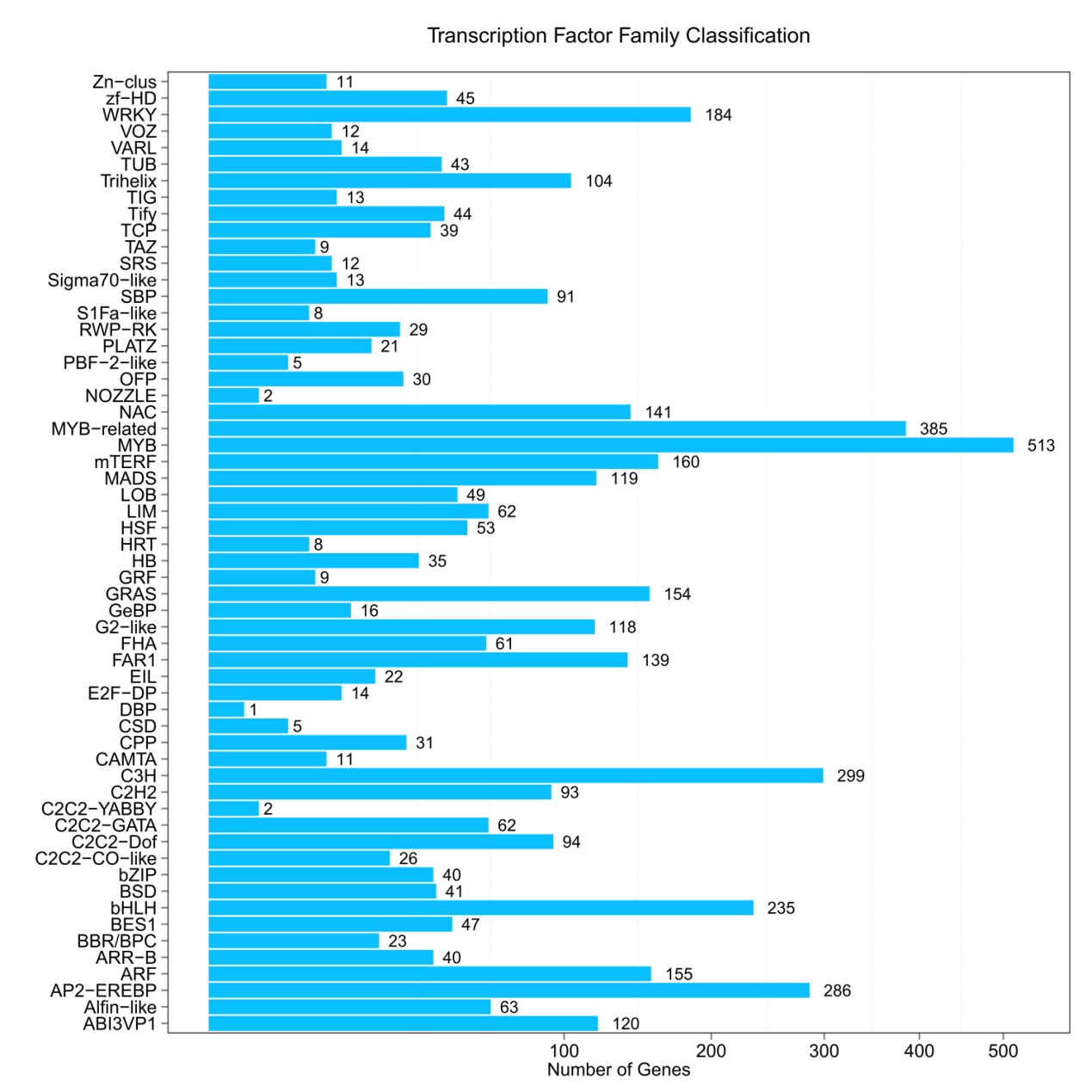
**

**Figure S6** Identified transcription factor families from *R. glutinosa* transcriptome
